# Obesity and inflammation: reduced cytokine expression due to resveratrol in a human *in vitro* model of inflamed adipose tissue

**DOI:** 10.3389/fphar.2015.00079

**Published:** 2015-04-14

**Authors:** Ivana Zagotta, Elitsa Y. Dimova, Klaus-Michael Debatin, Martin Wabitsch, Thomas Kietzmann, Pamela Fischer-Posovszky

**Affiliations:** ^1^Division of Pediatric Endocrinology and Diabetes, Department of Pediatrics and Adolescent Medicine, Ulm University Medical CenterUlm, Germany; ^2^Faculty of Biochemistry and Molecular Medicine, Biocenter Oulu, University of OuluOulu, Finland; ^3^Department of Pediatric and Adolescent Medicine, Ulm University Medical CenterUlm, Germany

**Keywords:** obesity, inflammation, cytokines, resveratrol, transcriptional activation, adipocytes, white

## Abstract

Obesity is associated with an inflammatory status and linked with a number of pathophysiological complications among them cardiovascular disease, type 2 diabetes mellitus, or the metabolic syndrome. Resveratrol was proposed to improve obesity-related inflammatory problems, but the effect of resveratrol on cytokine expression in obesity is not completely understood. In this study, we used an *in vitro* model of human adipose tissue inflammation to examine the effects of resveratrol on the production of the inflammatory cytokines interleukin 6 (IL-6), IL-8, and monocyte chemoattractant protein 1 (MCP-1). We found that resveratrol reduced IL-6, IL-8, and MCP-1 levels in a concentration-dependent manner in adipocytes under inflammatory conditions. Further experiments showed that the action of resveratrol was mainly due to its NFκB inhibitory potential. Thus, our data support the concept that resveratrol can alleviate obesity-induced up-regulation of inflammatory cytokines providing a new insight toward novel treatment options in obesity.

## Introduction

During the last 20 years obesity became a major public health problem in advanced and developing countries (De Ferranti and Osganian, 2007). Obesity is characterized by an excessive accumulation of white adipose tissue. While the total body composition of lean adult men consists of about 20% adipose tissue, the latter can increase to more than 40% in obese men. In addition to their fat storing and releasing capacity, white adipocytes represent endocrine cells that secrete a diverse range of adipokines and cytokines. Indeed, adipokines are involved in the regulation of various functions, including appetite, insulin sensitivity, angiogenesis, blood pressure, and the immune response (Maury and Brichard, 2010). As such, it is not surprising that cardiovascular disease, type 2 diabetes mellitus, the metabolic syndrome, and various cancers are ultimately linked to dyslipidemia, hyperglycemia, hypertension, and a pro-inflammatory state (De Ferranti and Osganian, 2007).

This endocrine function of the adipose tissue implicates that adipocytes communicate with other tissues and cells in a bidirectional manner. This appears to be central during development of an inflammatory state in the adipocyte tissue which upon excessive expansion is infiltrated by macrophages (Bouloumie et al., 2005). The infiltrated macrophages secrete inflammatory cytokines thereby leading to a local and systemic inflammation. In turn, this inflammation induces expression and secretion of cytokines like interleukin 6 (IL-6), IL-8, and monocyte chemoattractant protein 1 (MCP-1) from the adipocytes. Importantly, the levels of circulating IL-6, IL-8, and MCP-1 were shown to correlate with a higher body mass index (BMI; Herder et al., 2005; Warnberg et al., 2006; Derosa et al., 2013). Further, overproduction of IL-6 and MCP-1 by inflamed adipocytes could be directly linked with development of type 2 diabetes in obese patients where the paracrine action of MCP-1 decreases PPARγ expression thus leading to enhanced insulin resistance of adipocytes (Serrano-Marco et al., 2012). In addition, elevated plasma levels of IL-8 are associated with atherosclerosis and an increased risk of arterial plaque formation (Kobashi et al., 2005; Yang et al., 2006). When obese patients undergo calorie restriction, the levels of IL-6, IL-8, and MCP-1 appear to decrease implying that calorie restriction has a beneficial effect on the inflammatory state (Larson-Meyer et al., 2008).

Resveratrol, a natural polyphenolic compound, produced by plants in response to environmental stress and found in red grape skin, peanuts, a variety of berries and medical plants (Signorelli and Ghidoni, 2005) gained special interest as a calorie restriction mimetic based on data from rodents. When mice and/or rats were fed a high-fat diet, resveratrol treatment improved glucose homeostasis, mitochondrial function, lipid parameters, body weight, and survival (Baur et al., 2006; Lagouge et al., 2006; Barger et al., 2008; Pearson et al., 2008; Rivera et al., 2009; Rocha et al., 2009). While the resveratrol effects are intensively studied in animal models only few clinical trials were conducted so far to study the effects of resveratrol supplementation in the context of human obesity and coronary artery disease (Timmers et al., 2011; Tome-Carneiro et al., 2013b), yet there exists some controversy (Poulsen et al., 2013) and the effect of resveratrol on the expression of inflammatory cytokines, in particular IL-6, IL-8, and MCP-1 in obesity remains to be further investigated. Therefore it was the aim of this study to investigate the effects of resveratrol on the production of IL-6, IL-8, and MCP-1 in human adipocytes and in an *in vitro* model of human adipose tissue inflammation. We found that resveratrol reduces IL-6, IL-8, and MCP-1 levels in adipocytes under inflammatory conditions. Thus, our data support the concept that resveratrol can alleviate obesity-induced up-regulation of inflammatory cytokines in adipocytes.

## Materials and Methods

### Materials

All chemicals and reagents were obtained from commercial suppliers. Cell culture media and supplements were purchased from Invitrogen Life Technologies (Darmstadt, Germany). Resveratrol and LY294002 were obtained from Sigma (Deisenhofen, Germany), SC-514 was from Merck Millipore (Darmstadt, Germany). All the chemicals were diluted in DMSO which alone was used as a vehicle control. The following concentrations of the chemicals were used: for resveratrol, 10, 30, and 100 μM; for LY294002, 20 μM; for SC-514, 100 μM.

### Cell Culture

**FIGURE 1:**
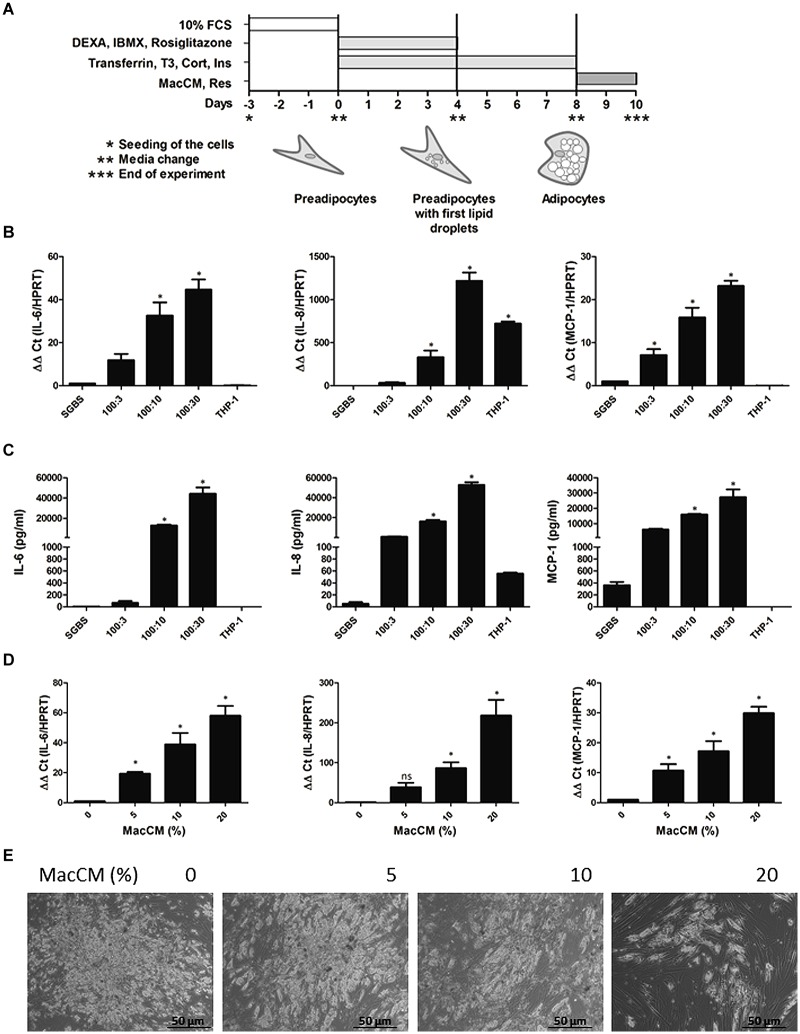
**Induction of interleukin 6 (IL-6), IL-8, and monocyte chemoattractant protein 1 (MCP-1) expression in an *in vitro* model of human inflamed adipose tissue. (A)** Scheme of the Simpson–Golabi–Behmel syndrome (SGBS) cell differentiation protocol. For details see “Materials and Methods.” **(B)** SGBS adipocytes were incubated for 48 h with increasing ratios of THP-1 macrophages. The mRNA levels were analyzed by qPCR and results were normalized to hypoxanthine phosphoribosyltransferase (HPRT). Data from three independent experiments are presented as mean ± SD. ^∗^*p* < 0.05 SGBS alone vs. SGBS:THP-1 or THP-1. **(C)** The accumulation of IL-6, IL-8, and MCP-1 in the media was measured by ELISA after coincubation with THP-1 cells for 24 h. **(D)** SGBS adipocytes were incubated with increasing doses of macrophage-conditioned media (MacCM) or vehicle for 48 h. The mRNA levels were analyzed by qPCR and results were normalized to HPRT. ^∗^Significant difference control vs. MacCM. **(E)** SGBS adipocytes were incubated for 48 h with increasing concentrations of MacCM or vehicle control. Representative images of SGBS cells after treatment are shown. **(E)** Scheme of the SGBS cell differentiation protocol. For details see “Materials and Methods.”

Human primary preadipocytes were prepared by collagenase digestion from subcutaneous adipose tissue of four healthy women using a previously described protocol (Hauner et al., 2001). Procedures were approved by the ethical committee of the University of Ulm and patients gave written informed consent. Simpson–Golabi–Behmel syndrome (SGBS) preadipocytes were cultured as described previously (Wabitsch et al., 2001; Fischer-Posovszky et al., 2008). These cells were used because they provide so far the only human preadipocyte model with high capacity for adipose differentiation. Adipogenic differentiation of SGBS and human primary preadipocytes was induced in serum-free DMEM/F12 medium supplemented with 10 μg/mL iron-poor transferrin, 10 nM insulin, 200 pM thyroid hormone, and 0.1 μM cortisol. For the first 4 days 2 μM rosiglitazone, 250 μM isobutylmethylxanthine, and 25 nM dexamethasone was added. Morphologically differentiated adipocytes were used for experiments 8 days after initiation of adipogenic differentiation (**Figure 1A**). The number of differentiated cells was estimated in the monolayers by direct counting using a net micrometer. SGBS cultures were used for experiments when differentiation rate was ≥85%.

THP-1 cells (ATCC, Wesel, Germany) were cultured as described earlier (Kotnik et al., 2013). Differentiation into macrophages was induced by 125 ng/mL phorbol-12 myristate 13-acetate (PMA) for 48 h. Macrophage-conditioned medium (MacCM) was collected after additional 48 h of incubation in serum-free basal medium containing 0.5% BSA and cleared by centrifugation. MacCM from five independently performed productions was pooled and then used for experiments. Serum-free basal medium containing 0.5% BSA was also used as a vehicle control at the corresponding concentrations.

Coculture experiments of SGBS adipocytes with THP-1 macrophages were performed as described before (Kotnik et al., 2013)*. In vitro* differentiated THP-1 macrophages were trypsinized and added to cultures of SGBS adipocytes (at day 9 of the adipogenic differentiation); different ratios between the SGBS and THP-1 cells were set up (SGBS:THP-1; 100:3, 100:10, 100:30). Treatment with resveratrol occurred for 48 h if not otherwise stated in the legends to the figures.

### Expression Analysis of mRNA by Quantitative Real-Time PCR (qRT-PCR)

Total RNA was prepared using the peqGOLD HP Total RNA kit (Peqlab, Erlangen, Germany) following the manufacturer’s instructions. One microgram of total RNA was used for cDNA synthesis with using SuperScript II Reverse Transcriptase (Invitrogen, Darmstadt, Germany). Quantitative real-time PCR (qRT-PCR) was performed with a LightCycler^TM^ 2.0 (Roche Diagnostics, Mannheim, Germany) using a LightCycler FastStart DNA Master PLUS SYBR Green I kit (Roche Diagnostics, Mannheim, Germany). The qRT-PCR results were normalized using hypoxanthine phosphoribosyltransferase (HPRT) as a housekeeping gene**.** The following primer sets were used: HPTR-forw (5′-GAGATGGGAGGCCATCACATTGTAGCCCTC-3′), HPRT-rev (5′-CTCCACCAATTACTTTTATGTCCCCTGTTGACTGGTC-3′), IL-6-forw (5′-TATACCCCCAGGAGAAGATTCC-3′), IL-6-rev (5′-TTTTCTGCCAGTGCCTCTTT-3′), IL-8-forw (5′-TGCCAAGGAGTGCTAAAGAACTTAGATGTCAG-3′), IL-8-rev (5′-AGCTTTACAATAATTTCTGTGTTGGCGCAGTG-3′), MCP-1-forw (5′-TCCCAAAGAAGCTGTGATCTTCAAGACC-3′), MCP-1-rev (5′-AGTGAGTGTTCAAGTCTTCGGAGTTTGG-3′). The experiments for each data point were carried out in triplicate. The relative quantification of gene expression was determined using the ΔΔCt method.

### ELISA

Protein secretion of IL-6, IL-8, and MCP-1 was measured in the supernatant. For determining the IL-6 and IL-8 concentration in the supernatant the commercially available IL-6, and IL-8 ELISA kits (BioSource, Nivelles, Belgium) were used according to the manufacturer’s protocols. The MCP-1 protein levels were determined using the Human CCL2 (MCP-1) ELISA Ready-SET-Go!^®^ kit (eBioscience, Frankfurt, Germany) following the manufacturer’s instructions.

### Statistics

Data are represented as mean ± SEM of three different experiments unless it is otherwise stated. Statistical significance was calculated by using one way analysis of variants (ANOVA) and Dunnet or Tukey correction test, where *p* < 0.05 was considered as statistically significant.

## Results

### The Cytokines IL-6, IL-8, and MCP-1 are Upregulated in an *In Vitro* Model of Human Inflamed Adipose Tissue

Obesity is associated with low grade chronic inflammation (Gregor and Hotamisligil, 2011) and in order to mimic this in human adipose tissue we first investigated whether direct cell interactions between macrophages and adipocytes have an impact on IL-6, IL-8, and MCP-1 expression. To do this, we used a coculture model in which we cocultured SGBS adipocytes directly with THP-1 macrophages in the following ratios (SGBS:THP-1; 100:3, 100:10, 100:30). Coculture of SGBS adipocytes with THP-1 cells increased IL-6, IL-8, and MCP-1 expression in a THP-1 cell-dependent manner. When the SGBS:THP-1 ratio was 100:30 the IL-6 mRNA was induced by about 45-fold, IL-8 mRNA was induced by about 1200-fold, and MCP-1 mRNA was induced by about 20-fold (**Figure 1B**). The mRNA expression was followed by the protein expression and increased IL-6, IL-8, and MCP-1 levels could be measured in the media. By contrast, THP-1 cells alone did not secrete significant amounts of IL-6, IL-8, and MCP-1 suggesting that adipocytes would be the source of the cytokines (**Figure 1C**).

Next we examined whether macrophage released mediators are able to cause an enhanced IL-6, IL-8, and MCP-1 expression in the adipocytes. Therefore, we incubated SGBS adipocytes with medium supplemented with increasing concentrations of MacCM for 48 h (Keuper et al., 2011). Next, we measured expression of IL-6, IL-8, and MCP-1 in SGBS adipocytes upon treatment with MacCM. MacCM induced a concentration-dependent up-regulation of IL-6, IL-8, and MCP-1 expression. While the presence of 5% MacCM already significantly increased IL-6, and MCP-1 mRNA levels by about 20-fold and 10-fold, respectively, 10% MacCM induced IL-6 mRNA by about 50-fold, IL-8 mRNA by about 90-fold and MCP-1 mRNA by about 20-fold compared to vehicle control (**Figure 1D**). Treatment of SGBS adipocytes with MacCM also induced morphological changes toward a more dedifferentiated fibroblast-like phenotype in a concentration-dependent manner. Especially higher concentrations of MacCM (20%) caused lipid loss from the adipocytes (**Figure 1E**) which was further confirmed by a reduced number of lipid filled adipocytes by morphological counting and an accumulation of free glycerol in the media supernatants suggesting a loss of lipids by lipolysis (data not shown) as shown earlier (Kotnik et al., 2013); therefore we conducted further experiments with 10% MacCM.

Together, these data show that macrophage released mediators induce IL-6, IL-8, and MCP-1 expression in adipocytes.

### Resveratrol Abolishes the Increased Expression of IL-6, IL-8, and MCP-1 in an *In vitro* Model of Human Inflamed Adipose Tissue

**FIGURE 2:**
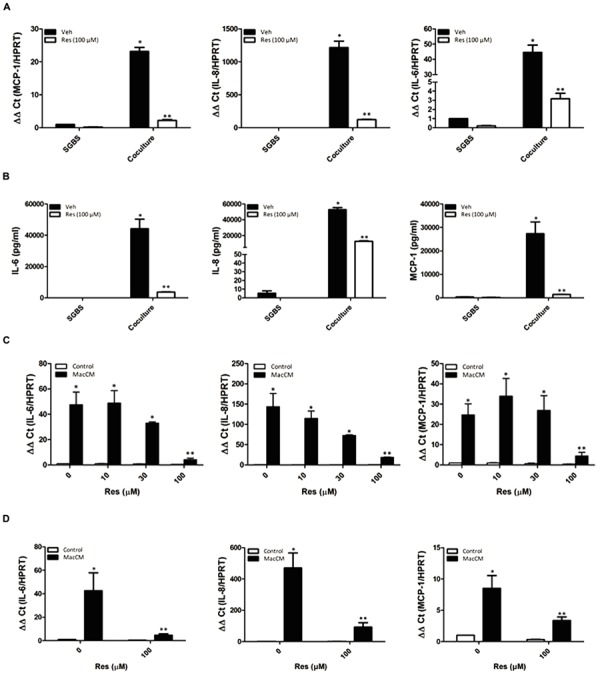
**Resveratrol (Res) abolished the macrophage and MacCM-dependent IL-6, IL-8, and MCP-1 induction in SGBS adipocytes and primary human *ex vivo* differentiated adipocytes. (A)** SGBS adipocytes were incubated for 48 h with THP-1 macrophages (100:30) and treated with 100 μM Res. The mRNA levels were analyzed by qPCR and results were normalized to HPRT. ^∗^Significant difference control vs. coculture (SGBS:THP-1; 100:30). ^∗∗^Significant difference coculture vs. coculture+Res **(B)** The accumulation of IL-6, IL-8, and MCP-1 in the media was measured by ELISA after coincubation with THP-1 cells and/or treatment with Res for 48 h. **(C)** SGBS adipocytes were incubated for 48 h with 10% MacCM or a combination of different doses of Res and 10% MacCM. The mRNA levels were analyzed by qPCR and results were normalized to HPRT. ^∗^Significant difference untreated vs. Res or MacCM, ^∗∗^Significant difference MacCM treated vs. MacCM +Res. **(D)** Primary human differentiated adipocytes were incubated for 48 h with 10% MacCM or 100 μM Res and 10% MacCM. The mRNA levels were analyzed by qPCR and results were normalized to HPRT. ^∗^Significant difference untreated vs. Res or MacCM, ^∗∗^Significant difference MacCM treated vs. MacCM +Res.

To determine the effect of resveratrol on the increased expression of IL-6, IL-8, and MCP-1, we first cocultured SGBS adipocytes directly with THP-1 macrophages (SGBS:THP-1; 100:30) in the presence or absence of resveratrol (100 μM). While coculture of SGBS adipocytes with THP-1 cells increased IL-6, IL-8, and MCP-1 expression resveratrol abolished the induced cytokine expression on the mRNA and protein level in the coculture model of inflamed adipose tissue (**Figures 2A,B**).

We next examined whether the resveratrol effect is also present in the MacCM treated SGBS adipocytes. Therefore, SGBS cells were cultured with 10% MacCM either in the absence or presence of different concentrations of resveratrol (10, 30, 100 μM) for 48 h. MacCM treatment induced IL-6 mRNA by about 50-fold, IL-8 mRNA by about 140-fold, and MCP-1 mRNA by about 25-fold. Treatment of SGBS adipocytes with MacCM and increasing doses of resveratrol resulted in a concentration-dependent reduction of cytokine expression when compared to MacCM treatment alone: IL-6 mRNA expression was reduced to about 10-fold, IL-8 mRNA to about eightfold and MCP-1 mRNA to about fivefold when treated with 100 μM resveratrol (**Figure 2C**).

Thus, these data indicate that resveratrol is able to reduce the inflammation associated expression of IL-6, IL-8, and MCP-1 in adipocytes.

### Resveratrol Abolishes the Increased Expression of IL-6, IL-8, and MCP-1 in Primary Adipocytes from Obese Patients

In order to demonstrate that the resveratrol effect is not limited to the SGBS cell model system of inflamed adipose tissue, we used human primary *ex vivo* differentiated adipocytes from four patients and incubated them with 10% MacCM in the presence or absence of resveratrol (100 μM) for 48 h. The IL-6 mRNA expression was induced by about 35-fold, the IL-8 mRNA was induced by about 320-fold, and MCP-1 mRNA expression was induced by about sevenfold. Treatment with resveratrol diminished the MacCM-dependent expression of IL-6, IL-8, and MCP-1 to sixfold, eightfold, and twofold, respectively (**Figure 2D**).

### PI3K Inhibition Only Affects MCP-1 but Not IL-6 and IL-8 Expression

**FIGURE 3:**
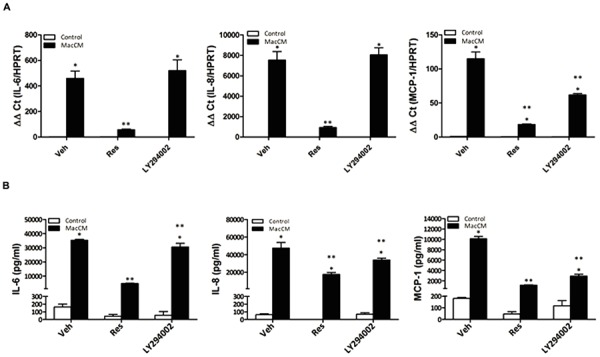
**The effects of Res on MCP-1 but not IL-6, and IL-8 gene expression in SGBS adipocytes are mediated via PI3K. (A)** Where indicated SGBS adipocytes were treated with a combination of 10% MacCM and 20 μM LY294002, or 100 μM Res for 48 h. The mRNA levels were analyzed by qPCR and results were normalized to HPRT. ^∗^Significant difference control vs. treated ^∗∗^significant difference MacCM vs. MacCM +Res or MacCM+LY204002. **(B)** The accumulation of IL-6, IL-8, and MCP-1 in the media was measured by ELISA after treatment with 10% MacCM and/or 20 μM LY294002, 100 μM Res for 48 h. ^∗^Significant difference control vs. treated ^∗∗^significant difference MacCM vs. MacCM +Res or MacCM + LY204002.

Resveratrol has been shown to affect several pathways from which the PI3K/Akt pathway (Kang et al., 2010; Mader et al., 2010; Miranda et al., 2010; Wang et al., 2011) appears to be of importance in an inflammatory setting. To examine whether PI3K is involved in the resveratrol-dependent inhibition of IL-6, IL-8, and MCP-1 gene expression, we incubated SGBS adipocytes with the PI3K inhibitor LY294002 (20 μM) along with resveratrol (100 μM), MacCM (10%) or their combinations and determined IL-6, IL-8, and MCP-1 mRNA levels 48 h after treatment. While resveratrol decreased IL-6, IL-8, and MCP-1 mRNA levels by more than 80% compared to MacCM treatment alone, treatment with LY294002 decreased only MCP-1 mRNA and protein levels by 50% and by about 70%, respectively, but did not decrease IL-6 and IL-8 mRNA expression; on protein level it decreased IL-6 expression by 13% and IL-8 by 28%. Thus, these data indicate that the action of resveratrol on IL-6 and IL-8 expression is rather not PI3K pathway dependent (**Figure 3**).

### Resveratrol Abolishes the Increased Expression of IL-6, IL-8, and MCP-1 by Acting Like an NFκB Inhibitor

**FIGURE 4:**
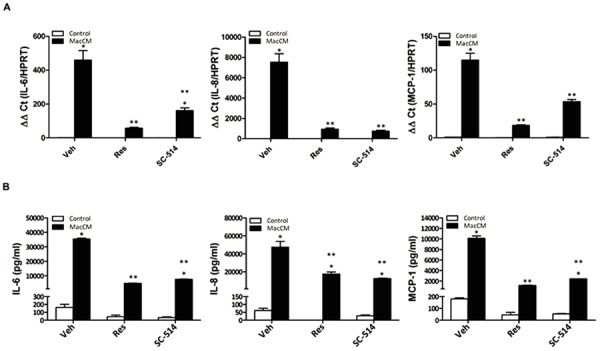
**Resveratrol acts like an NFkB inhibitor suppressing the MacCM-dependent IL-6, IL-8, and MCP-1 gene expression. (A)** SGBS adipocytes were treated with 10% MacCM, a combination of both Res (100 μM) and 10% MacCM, or a combination of SC-514 (100 μM) and MacCM for 48 h. The mRNA levels were analyzed by qPCR and results were normalized to HPRT. ^∗^Significant difference control vs. treated ^∗∗^significant difference MacCM vs. MacCM +Res or MacCM+SC-514. **(B)** The accumulation of IL-6, IL-8, and MCP-1 in the media was measured by ELISA after treatment with 10% MacCM and/or 100 μM SC-514, 100 μM Res for 48 h. ^∗^Significant difference control vs. treated ^∗∗^significant difference MacCM vs. MacCM +Res or MacCM+SC-514.

The reduction of IL-6, IL-8, and MCP-1 expression by resveratrol under inflammatory conditions may be partially explained by the ability of resveratrol to suppress the activity of NFκB, a transcription factor critically involved in inflammation. We have shown previously that resveratrol is able to inhibit nuclear translocation of NFκB in SGBS adipocytes (Zagotta et al., 2013). Therefore, we further explored the role of NFκB on IL-6, IL-8, and MCP-1 production in SGBS adipocytes treated with MacCM, resveratrol, and the NFκB inhibitor SC-514 alone or in combination. The expression of IL-6, IL-8, and MCP-1 was assayed by qPCR and ELISA. The treatment with the NFκB inhibitor SC-514 reduced MacCM-dependent IL-6, IL-8, and MCP-1 induction (**Figure 4A**), Further, resveratrol exhibited a strong reduction on the MacCM-dependent IL-6, IL-8, and MCP-1 induction on the mRNA and protein level. Together, these data suggest that resveratrol inhibits the MacCM-dependent IL-6, IL-8, and MCP-1 induction at least to a large extend via its NFκB inhibitory potential (**Figure 4**).

## Discussion

In this study we investigated the human IL-6, IL-8, and MCP-1 expression in a model of inflamed human adipose tissue. Our data demonstrated several new findings on the expression of IL-6, IL-8, and MCP-1 under obesity-mimicking conditions. First, it was found that macrophage-released mediators and not direct cell contacts are responsible for the enhanced expression of IL-6, IL-8, and MCP-1 in SGBS adipocytes. Second, resveratrol strongly inhibited the obesity-associated and inflammation-dependent induction of IL-6, IL-8, and MCP-1 in SGBS adipocytes. Third, resveratrol acted to a large extend like an NFκB inhibitor but less like a PI3K inhibitor on the inflammatory-dependent IL-6, IL-8, and MCP-1 expression.

Obesity represents a risk factor for the development of diseases like type 2 diabetes mellitus, hypertension, atherosclerosis, and myocardial infarction. Intriguingly, obesity is also associated with a state of chronic low-grade inflammation characterized by elevated plasma concentrations of pro-inflammatory cytokines (IL-6, IL-1, and TNF-α), chemokines (MCP-1) and adipokines (haptoglobin, PAI-1, leptin, visfatin, resistin, and VEGF; Hotamisligil, 2006). Plasma IL-6, IL-8, and MCP-1 levels are considerably enhanced in obese humans and in patients with insulin resistance, type 2 diabetes, and cardiovascular diseases (Skurk et al., 2002; Lyon and Hsueh, 2003). It is estimated that about 35% of obesity-related IL-6 in serum is secreted from adipose tissue (Mohamed-Ali et al., 1997). Indeed, the present data in accordance with previous reports (Path et al., 2001; Vicennati et al., 2002; Ajuwon et al., 2004; Deo et al., 2004; Ajuwon and Spurlock, 2005; Fain and Madan, 2005) indicate that adipocytes can produce substantial amounts of IL-6, IL-8, and MCP-1.

Although all these data indicate that the adipocytes appear to be the major source of elevated IL-6, IL-8, and MCP-1 levels observed in obesity (Shimomura et al., 1996; Morange et al., 1999), it is still a matter of debate whether this is a result of stimulation of adipocytes via direct cell contacts with macrophages or an effect caused by hormones and cytokines released from the macrophages (Loskutoff and Samad, 1998). The current study shows that direct cell contacts between THP-1 macrophages and adipocytes are of less importance since incubation of adipocytes with only the THP-1 conditioned medium was enough to cause enhanced IL-6, IL-8, and MCP-1 expression. In turn, the THP-1 released cytokines present in the MacCM, in particular TNFα, may play a role in the lipolysis observed in the current study.

New aspects in obesity treatment consider to reduce the inflammatory burden and to reduce cytokine expression in adipocyte by use of natural compounds. Resveratrol is such a compound which is at least capable to change the obesity-associated secretion profile of adipocytes (Ahn et al., 2007; Olholm et al., 2010; Yen et al., 2011; Rosenow et al., 2012). In particular resveratrol inhibited TNF-α-dependent upregulation of the acute phase protein PAI-1 in 3T3-L1 adipocytes (Ahn et al., 2007; Olholm et al., 2010; Yen et al., 2011; Rosenow et al., 2012), and PAI-1 production in human SGBS adipocytes (Rosenow et al., 2012; Zagotta et al., 2013). These data are very much in line with the results from the present study where we have shown that resveratrol exerted a very pronounced effect on IL-6, IL-8, and MCP-1 expression in a model of inflamed human adipose tissue (**Figures 2** and **3**). Although all these data indicate that resveratrol can alleviate obesity-induced upregulation of IL-6, IL-8, and MCP-1 in adipose tissue, it has not been fully elucidated by which molecular mechanisms resveratrol exerts its effect on IL-6, IL-8, and MCP-1 under inflammatory conditions. Resveratrol is known to exert pleiotropic effects on cells via Sirt1 (Picard et al., 2004; Fischer-Posovszky et al., 2010), AMPK (Wang et al., 2011; Lasa et al., 2012), and PI3K/Akt (Kang et al., 2010; Mader et al., 2010; Miranda et al., 2010) and nuclear factor (NF) κB. However, our recent investigation with SGBS adipocytes has shown that Sirt1, and AMPK were not the major mediators of the resveratrol effects on PAI-1 synthesis under inflammatory conditions (Zagotta et al., 2013). Indeed a number of studies indicated that the PI3K/Akt pathway represents an important signaling cascade in the initiation of the inflammatory response. Although we showed in an earlier study that resveratrol inhibits PI3K-driven Akt phosphorylation in SGBS cells (Mader et al., 2010) like the PI3K inhibitor, LY294002, the present study shows that LY294002, in contrast to resveratrol, could not abrogate the MacCM-dependent up-regulation of IL-6, and IL-8. LY294002 had only an effect on MCP-1 (**Figure 3B**) implicating that inhibition of the PI3K/Akt pathway by resveratrol is not involved in the attenuating effect on MacCM-dependent IL-6, IL-8, and MCP-1 expression. The reason for this different pattern is not entirely clear. One option could be that the effect of PI3K inhibition on MCP1 was more direct via a PI3K or Akt regulated transcription factor whereas it was rather indirect or involving PI3K regulated feed back circuits with IL6 and IL8. For example if there are some suppressors of IL6 and IL8 expression which are regulated by PI3K, their inhibition by PI3K inhibitors would have a positive effect on IL6 and IL8 expression. Overall, the net result would be that PI3K inhibition has no effect on IL6 and IL8 expression.

An increase in plasma IL-6, IL-8, and MCP-1 levels observed in obesity can also be the result of a cytokine-dependent induction of NFκB, a transcription factor with a central role in the induction of a chronic inflammatory state associated with obesity, development of type 2 diabetes, cardiovascular risk, and insulin resistance (Gonzales and Orlando, 2008). Previous reports including our own indicated that resveratrol can act as an inhibitor of NFκB (Kundu and Surh, 2004; Tome-Carneiro et al., 2013a). The data of the present study showing that resveratrol behaves like an established NFκB inhibitor and reduces more or less completely the MacCM-dependent expression of IL-6, IL-8, and MCP-1 are in line with those findings. This places NFκB in a central position to be the mediator of obesity-associated inflammatory effects and links a number of *in vivo* and *in vitro* studies showing an inhibitory role of the resveratrol targets Sirt1, and AMPK on NFκB (Pfluger et al., 2008; Yoshizaki et al., 2009; Zhu et al., 2011; Lin et al., 2012; references in Salminen et al., 2011). Although several previous findings have demonstrated that the PI3K/Akt pathway has a crucial role in the activation of the NFκB pathway (Ozes et al., 1999; Madrid et al., 2001) our current data suggest that the PI3K/Akt pathway is less dominantly involved in the MacCM-dependent expression of the IL-6, and IL-8 genes.

Together, our current findings add at least one novel aspect to the pleiotropy of the resveratrol effects by showing that it can act as an anti-inflammatory molecule blocking the NFκB-dependent expression of the inflammatory cytokines IL-6, IL-8, and MCP-1 in an *in vitro* model of human inflamed adipose tissue. These findings may be useful to further establish IL-6, IL-8, and MCP-1 as markers for obesity-associated inflammatory conditions.

## Conflict of Interest Statement

The authors declare that the research was conducted in the absence of any commercial or financial relationships that could be construed as a potential conflict of interest.
